# Incidence and comparison of retrospective and prospective data on respiratory and gastrointestinal infections in German households

**DOI:** 10.1186/s12879-017-2434-5

**Published:** 2017-05-11

**Authors:** Kristin Maria Schlinkmann, Abhishek Bakuli, Rafael Mikolajczyk

**Affiliations:** 1 Department for Epidemiology, Helmholtz Centre for Infection Research, ESME – Epidemiological and Statistical Methods Research Group, Inhoffenstraße 7, 38124 Braunschweig, Germany; 2 PhD Programme “Epidemiology”, Braunschweig-Hannover, Germany; 3 Hannover Medical School, Hannover, Germany; 4 German Centre for Infection Research Site (DZIF), Braunschweig-Hannover, Germany; 5 Institute for Medical Epidemiology, Medical Faculty of the Martin Luther University Halle-Wittenberg, Biometrics, and Informatics (IMEBI), Halle (Saale), Germany

**Keywords:** Incidence, Gastrointestinal infections, Respiratory infections, Study design

## Abstract

**Background:**

Acute respiratory infections (ARI) and acute gastrointestinal infections (AGI) are the most common childhood infections, and corresponding data can either be collected prospectively or retrospectively. The aim of this study was to estimate the incidence of respiratory and gastrointestinal episodes in German households with children attending day care and to compare results of prospective and retrospective data collection.

**Methods:**

We conducted a 4 months prospective cohort study in the winter period 2014/2015 and recruited parents of children aged 0–6 years in 75 day care centers in Braunschweig, Lower Saxony, Germany. For all household members, we collected information on episodes of ARI and AGI. We applied prospective data collection in one study arm and retrospective data collection with a reporting period of 2 months in the other. Poisson regression was used to model monthly incidence rates for both study arms.

**Results:**

In total, 100 households (including 404 persons) participated in the retrospective group and 77 households (282 persons) in the prospective group. Incidence estimates for ARI (retrospective group: 0.52 per person month, prospective group: 0.47) were higher than for AGI (retrospective group: 0.14, prospective group: 0.13). The adjusted incidence estimates were similar in both study arms for ARI (incidence rate ratio for retrospective versus prospective data collection: 1.11 [confidence interval (CI) 95% 0.99; 1.24], *p* = 0.42) as well as for AGI (1.10 [CI 95% 0.89; 1.37], *p* = 0.27).

**Conclusion:**

If there is no need to collect biomaterials or data on severity of the diseases, incidence of infections in the household setting over a short time period (2 months) can be assessed retrospectively.

**Electronic supplementary material:**

The online version of this article (doi:10.1186/s12879-017-2434-5) contains supplementary material, which is available to authorized users.

## Background

Acute respiratory infections (ARI) and acute gastrointestinal infections (AGI) are the most common childhood infections, and they cause significant burden and costs in the health care system [1, 2]. Other household members and especially parents are at risk to frequently acquire these infections from their children [3]. In consequence, households including children have a higher risk of suffering from infectious diseases, especially in winter seasons. Roy et al. [4] published a systematic review of 33 studies with various designs on incidence of AGI in developed countries and reported an incidence, which varies between 0.1 and 3.2 infections per person year. For ARI, an Australian study has reported an incidence of 3.2 cases per person year in the general population [5]. Also, ARI frequency is known to be higher in households with children aged younger than 5 years than in other households [6].

Data on both types of infections can either be collected prospectively or retrospectively. Whereas the latter approach may be biased due to recall problems [7], prospective data collection can be very complex, time consuming, and costly [8]. Besides, loss to follow up is a further disadvantage of prospective studies, which may occur already at 1 month of follow up [9]. Recently, Viviani et al. [10] compared the incidence estimates of AGI in their retrospective telephone survey with another study with prospective data collection during the same time period [11], and found higher incidence estimates in the retrospective data collection for the general population. But publications explicitly comparing prospective and retrospective data collection for AGI in the households with children attending day care are scarce. Furthermore, no publications comparing both forms of data collection in households with children are available for ARI. Therefore, the aim of this study was to estimate the incidence of respiratory and gastrointestinal episodes in German households with children attending day care and to compare prospective and retrospective data collection in this setting.

## Methods

### Recruitment and study population

We conducted a 4 month prospective cohort study in the winter period 2014/2015 and recruited participants in 75 of 151 day care centres (DCCs) in Braunschweig, Lower Saxony, Germany. Initially, we contacted all DCCs in Braunschweig via a letter and a subsequent call. Around 50% of the contacted DCCs agreed to participate. In some of these DCCs, we got permission to invite parents to our study when they came to pick up their children from the DDC or during organisational meetings of the parents; in others, we only provided them flyers and these were distributed by the child care workers. We invited parents of children aged 0–6 years with their whole households for participation. Data on incidence of infections was collected between November 2014 and March 2015 for all household members. Participants in one study arm used a prospective health diary for documentation of infections for the whole study period, while those in the second study arm reported infections retrospectively. Participants could opt for participation in either study arm; the prospective data collection additionally included collection of nasal swabs in case of respiratory symptoms.

### Procedures

In the prospective study arm, one parent filled infection episodes for all household members in the diary, including start and end of each episode. Participants in the retrospective study arm reported the total number of infections for every household member in the last 2 months in questionnaires provided at 2 months and 4 months after beginning of the study. Definitions of ARI (based on Lambert et al. [12, 13]) and AGI (based on WHO definition of diarrhoea [14]) were provided in both groups at the start of the study (see Additional file 1 for details). The definition of a new episode required at least 3 consecutive days without symptoms following the previous episode. Socioeconomic data on household level and demographic data on all household members were also collected.

### Ethics statement

The study protocol was approved by the Ethics Committee of Hannover Medical School (No. 2380–2014) and reviewed by the Federal Commissioner for Data Protection and Freedom of Information. Written informed consent was obtained from all participants.

### Statistical analysis

We used tabulation to describe the study population and performed chi-squared test as well as Wilcoxon rank-sum test to assess differences between the two study groups (*p*-value < 0.05). Poisson regression was used to model monthly (30 days) incidence rates with an offset (person days observed) to adjust the denominator in the rate calculations and allowing for over-dispersion (using quasipoisson distribution for the generalized linear model (GLM)) [15]. The covariates of interest were the variables indicating the month and the study arm. The offset for the prospective group were the actual number of days observed for the individual in a given month. In contrast, for the retrospective group, total days in the respective month were used as offset. In the retrospective group, the exact date of onset of the disease was not known. Therefore, for the analysis considering specific months, we had to randomly assign the disease episodes to the 2 months before the return of the questionnaire. We chose an allocation scheme giving equal probability weights for 2 months, if the questionnaire was received in the first half of the month (½ and ½ for the two previous months respectively). When the questionnaire was received in the second half of the month, we allocated the episodes across 3 months giving double probability weight to the middle month, and equal weight to the remaining two half months in consideration (¼, ½, and ¼ for the 3 months, respectively). We also allocated questionnaires that arrived in the second half of April to 3 months (¼ February, ½ March and ¼ April), but since the prospective study was only conducted until March, we deleted the observations for April (10 ARI episodes and 2 AGI episodes). Based on observations from previous studies that participants might be more likely to join the study if they currently have an infection [16], we removed initial observations in the prospective arm, if the participant reported symptoms for these days, so that every individual started the observation with a healthy day. In total, this led to a decrease of 19% in the number of ARI episodes and 2% in the number of AGI episodes for November and December. Since infection episodes might also have triggered participation in the retrospective arm of the study, we removed the same fraction of infection episodes as in the prospective study arm. This was done by removing randomly the episodes from the first questionnaires, which would be potentially allocated to November and December in the retrospective group. The removal was done according to the age specific reduction that was observed in the prospective group due to starting with a healthy day for every individual. For ARI, the age group specific episode reduction was 9%, 11%, 13%, and 9% in the age groups 0–3 years, 4–6 years, 7–15 years and over 15 years, respectively. For AGI, the age group specific episode reduction was 4% and occurred only in the group of 0–3 years.

In a sensitivity analysis, equal probability weights (1/3, 1/3, and 1/3) were used for all 3 months when the questionnaire arrived in the second half of a month. When the questionnaire arrived in the first half of the month, the initially used probability weights remained unchanged. In a further sensitivity analyses, we removed the observations for the first 5 days for every individual in the prospective group, similar to Bayer and colleges [16], and proportionally decreased the infection episodes from the retrospective group in a similar way as described for the original comparison. Incidence rates per person year have additionally been calculated for comparison with existing literature.

## Results

### Study population

Approximately 4300 children attended the 75 DCCs included in the study, but due to different recruitment strategies, we do not know the exact number of parents invited to participate. In total, 341 families indicated interest to participate in our study. Of those, 95 families finally agreed to participate in the prospective group and 108 families in the retrospective group. Health diaries were returned from 77 households (with 282 household members), and retrospective questionnaires on the frequency of infections from 100 households (404 household members). In the prospective group, participants provided data for 32,739 days (nearly 100%) out of 32,798 expected days; in the retrospective group, 180 questionnaires were returned (90% of first questionnaires, 86% of second questionnaires). The median household size was four, and in most of these households at least one parent had a higher education (university degree). There were no differences in terms of parental education or age and sex composition of the households between the prospective and the retrospective study groups (Table 1).

**Table 1:** Description of the two study groups

	Prospective study group		Retrospective study group		Significance (*p*-value for differences between the study arms)
Median household size (interquartile range (IQR))	4 (3; 4)		4 (3; 5)		*p* = 0.08
	N	%	N	%	
Household level	77	100.0	100	100.0	
Highest education of the parents					
Professional or university training	59	76.6	78	78.0	*p* = 0.47
Max. intermediate level of vocational or secondary education	17	22.1	17	17.0	
Missing information	1	1.3	5	5.0	
Household members level	282	100.0	404	100.0	
Age					
0–3 years	63	22.3	84	20.8	*p* = 0.48
4–6 years	49	17.4	57	14.1	
7–15 years	16	5.7	28	6.9	
16 years and older	150	53.2	234	57.9	
Missing information	4	1.4	1	0.3	
Sex					
Female	146	51.8	197	48.8	*p* = 0.79
Male	134	47.5	206	51.0	
Missing information	2	0.7	1	0.3	

### Incidence of ARI and AGI episodes

Altogether, 1320 episodes of ARI were reported: 558 in the prospective and 762 in the retrospective group. For AGI, there were in total 331 episodes reported: 146 episodes in the prospective group and 185 in the retrospective group. After excluding episodes at the beginning of the records, 507 ARI episodes and 144 AGI episodes remained in the prospective group. After applying the proportional correction in the retrospective group, there were 691 ARI episodes and 183 AGI episodes remaining (Table 2).

**Table 2:** Incidence of acute respiratory (ARI) and gastrointestinal (AGI) infections, by mode of data collection and age group

		Prospective data collection				Retrospective data collection				Significance (p-value for differences between the study arms
		Person days	Episodes	Incidence per person month (30 days)^c^	Incidence per person year (365 days)	Person days	Episodes	Incidence per person month (30 days)^c^	Incidence per person year (365 days)	α = 0.05
ARI	Age groups									
	0–3 years	7009	164	0.7 (0.59; 0.82)	8.54	8311	217	0.78 (0.68; 0.89)	9.49	*p* = 0.31
	4–6 years	5729	100	0.52 (0.43; 0.64)	6.37	5882	103	0.53 (0.43; 0.64)	6.39	*p* = 1.00
	7–15 years	1891	13	0.21 (0.12; 0.33)	2.51	2828	41	0.43 (0.31; 0.59)	5.29	*p* = 0.02
	> 15 years	17,274	229	0.4 (0.35; 0.45)	4.84	22,970	330	0.43 (0.39; 0.48)	5.22	*p* = 0.37
	Total	32387^a^	507	0.47 (0.42; 0.52)	5.71	40050^b^	691	0.52 (0.48; 0.56)	6.32	*p* = 0.10
AGI	Age groups									
	0–3 years	7117	48	0.2 (0.16; 0.26)	2.46	8120	53	0.2 (0.15; 0.26)	2.37	*p* = 0.95
	4–6 years	5792	24	0.12 (0.08; 0.18)	1.51	5721	30	0.16 (0.11; 0.22)	1.91	*p* = 0.47
	7–15 years	1902	6	0.09 (0.04; 0.20)	1.15	2724	17	0.19 (0.11; 0.30)	2.28	*p* = 0.21
	> 15 years	17,441	66	0.11 (0.09; 0.15)	1.38	22,370	83	0.11 (0.09; 0.14)	1.35	*p* = 0.97
	Total	32736^a^	144	0.13 (0.11; 0.16)	1.61	38994^b^	183	0.14 (0.12; 0.16)	1.7	*p* = 0.60

^a^Missing information on age for 4 persons (contributing 484 person days), ^b^Missing information on age for 1 person (contributing 59 person days), ^c^Confidence Intervals (CI’s) in brackets

**Fig. 1:**
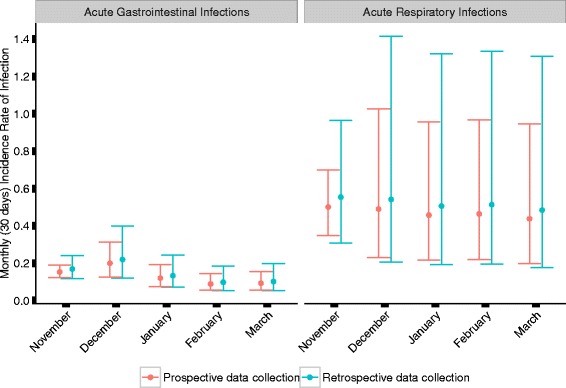
Incidence rates per person months for respiratory (ARI) and gastrointestinal infections (AGI) and the confidence intervals for the prospective and retrospective study types (adjusted model estimates)

The incidence for ARI varied by calendar month and ranged from 0.44 per person month to 0.54 in the retrospective group and from 0.36 to 0.63 in the prospective group. The mean monthly incidence for the retrospective group was 0.52 as compared to 0.47 for the prospective group. Incidence of ARI showed a decrease with increasing age of participants for both study arms, but was still substantial among adults (Table 2). After adjusting for the calendar month, the retrospective study arm was associated with a similar incidence as the prospective study arm (incidence rate ratio (IRR) for retrospective versus prospective data collection 1.11 [95% confidence interval (CI) 0.99; 1.24], *p* = 0.42) (Fig. 1).

The incidence for AGI also varied across the calendar months, ranging from 0.10 to 0.20 per person month in the retrospective study arm, and 0.08 to 0.22 in the prospective study arm. The mean monthly incidence for the retrospective group was 0.14 as compared to 0.13 in the prospective group. Also for AGI, the age specific incidence showed a general decrease with increasing age of children, with still substantial incidence among adults (Table 2). After adjusting for the calendar month, the prospective study arm was associated with a similar incidence as in the retrospective study arm (IRR 1.10, [95% CI 0.89; 1.37], *p* = 0.26) (Fig. 1).

Interestingly, the incidence estimates for all age groups were comparable between the prospective and the retrospective study group, except for participants in the age group “7–15 years”. They had an approximately 50% lower incidence in the prospective group compared to the retrospective group for ARI as well as for AGI, but the result was only statistically significant for ARI (*p* = 0.02).

Young children (0–3 years) had the highest incidence of infection (for both AGI and ARI), with similar results in the prospective and the retrospective group (IRR for the retrospective group with respect to the prospective group, adjusted for the age groups, was 1.11 ([95% CI 0.96; 1.28], *p* = 0.26) for ARI, and 1.07 ([95% CI 0.87; 1.31], *p* = 0.59) for AGI).

For all sensitivity analyses, the results with respect to the data collection mode were similar for both, ARI and AGI, and did not show a statistically significant difference between the two study groups (prospective versus retrospective).

## Discussion

In a longitudinal study on ARI and AGI in households with preschool children comparing prospective and retrospective data collection, we demonstrated that for short recall periods (2 months) both designs produce similar incidence estimates.

As expected, AGI had a lower incidence in our cohort than ARI [17]. While children had a higher incidence for ARI and AGI, there was also a substantial burden among older household members. For AGI, our incidence estimates over all age groups are consistent with the previously reported data by Roy et al. (0.1–3.2 per person year) [4]. In our study, we did not include fever as a symptom for ARI, what might lead to lower overall incidence estimates. Different disease definitions for ARI are used in literature, e.g. including fever as a defining symptom [12, 13, 18, 19] or not [20–22]. Use of different definitions can lead to considerable differences in the reported incidence [23]. Our results on the incidence (per person year) of ARI (5.7 in the prospective and 6.5 in the retrospective arm) were higher than obtained in an Australian study by Chen and Kirk (3.2) in the general population [5]. The higher incidence in our study can be explained by a higher proportion of families with at least one child attending day care, because these children have a higher risk for respiratory infections [24–26]. It was also observed that households with children have a general higher risk for respiratory infections [6]. Moreover, we conducted our study during the main season for respiratory infections in the Northern hemisphere (November–April).

Studies focusing on the comparison between prospective and retrospective data collection are scarce. In 2014, van der Steen and colleagues [27] called for more methodologic studies in the context of dementia research, which compare prospective and retrospective data collection. The recent study by Viviani et al. [10] found higher incidence estimates for gastrointestinal infections in the retrospective design in studies using recall periods of 7 and 28 days, compared to a prospective study during the same time period. Recall intervals in our study were even longer (2 months) than in the study by Viviani et al. (7 or 28 days) and might therefore not be comparable. We used a more specific definition for AGI in our study (Additional file 1) than the one in the UK, additionally contributing to the insufficient comparability. But despite these differences, we did not find a lower overall incidence of gastrointestinal infections in our study. However, as mentioned in the context of ARI, we conducted our study during the winter months and included only households with children attending day care, elucidating higher overall incidences. Regarding the recall intervals, Viviani et al. also found that the frequency of disease reports was a lot lower in their 28-days recall group than in their 7-days recall group (rate ratio comparing incidence in 7-day and 28-day recall groups: 2.9) [10]. This difference might be explained by people forgetting illness with time, as it was already shown by others [28]. As we used a recall period of 2 months, it might explain why our retrospective results are not significantly higher than our prospective results. We did not find any comparable study which compares the mode of data collection in terms of ARI. In general, the incidence of ARI is a lot higher than for AGI, but AGI episodes result in a greater burden of health care resources [17]. But this is another comparison, requiring a separate investigation, which was not conducted in our study.

To our knowledge, our study was the first of its kind, investigating the differences between prospective and retrospective data collection for both, respiratory or gastrointestinal infectious diseases in the household setting. However, our study was subject to some limitations: Participants in our study were not randomly allocated, but could opt for either the retrospective or the prospective mode of data collection. Since the prospective data collection included sampling of biomaterials, one could expect that those more dedicated opted for the prospective data collection. Still we did not observe a higher incidence in this group and did not find differences between the two study groups regarding socio-demographic characteristics. Another point is that the participants in our study knew in advance that they will receive a questionnaire on the frequency of infections after 2 and after 4 months of the study period, which could enhance their reporting. Results might be different in simple one time surveys, where participants are asked to report previous episodes without announcing the inquiry beforehand, because they might remember previous diseases as more recent and report them incorrectly in the enquired time period [29]. Furthermore, we did not study the question if symptom severity can be also recalled over the recall period, so if this information is required, additional investigation is necessary.

## Conclusion

In conclusion, retrospective (over 2 months) and prospective data collection in our study resulted in similar incidence estimates in case of ARI and AGI. Considering that participants will be informed about coming future recall periods, retrospective data collection might be an adequate method in the investigated setting, if the recall intervals are not too long and no need for bio sampling or details on the severity of diseases exists.

## Additional file

Additional file 1: Definition of acute respiratory (ARI) and gastrointestinal infection (AGI) episodes. (DOCX 19 kb)

## Abbreviations

AGI: Acute gastrointestinal infections
ARI: Acute respiratory infections
CI: Confidence interval
DCC: Day care center
GLM: Generalized linear model
IRR: Incidence rate ratio
