# Supplementary material for: Incidence and comparison of retrospective and prospective data on respiratory and gastrointestinal infections in German households
Source: BMC Infect Dis. 2017 May 11;17:336. doi: 10.1186/s12879-017-2434-5 (PMC5426066; doi:10.1186/s12879-017-2434-5)
Supplement: Additional file 1: — Definition of acute respiratory (ARI) and gastrointestinal infection (AGI) episodes. (DOCX 19 kb) [file 12879_2017_2434_MOESM1_ESM.docx]

Additional file 1

| **Disease** | **Definition** |
| --- | --- |
| **ARI**  (based on Lambert et al. [13, 12]) | Either **one** of the below stated symptoms of **category A**  **and/or**  **at least two** (or more) symptoms of **category B**.   \| Category A:   - Wheezing - Cough with sputum - Diagnosed otitis media or pneumonia \| Category B:   - Cold - Cough - Sore throat - Pain in the limbs - Headache - Shivering - Weakness/fatigue/decreased activity - Irritability - Vomiting \| \| --- \| --- \| |
| **AGI**  (based on definition of diarrhoea of WHO [14]) | At least **3-times** liquid/pasty stool per day (24h)  **and/or**  at least **one time** vomiting per day (24h). |
